# Novel biomarker *SYT12* may contribute to predicting papillary thyroid cancer outcomes

**DOI:** 10.4155/fsoa-2017-0087

**Published:** 2017-09-22

**Authors:** J Jonklaas, SRK Murthy, D Liu, J Klubo-Gwiezdzinska, J Krishnan, KD Burman, L Boyle, N Carrol, E Felger, Y P Loh

**Affiliations:** 1 Division of Endocrinology, Georgetown University, Washington, DC 20007, USA; 2 Section on Cellular Neurobiology, Eunice Kennedy Shriver National Institute of Child Health & Human Development, National Institutes of Health, Bethesda, MD 20892, USA; 3 Division of Intramural Population Health Research, Biostatistics & Bioinformatics Branch, Eunice Kennedy Shriver National Institute of Child Health & Human Development, National Institutes of Health, Bethesda, MD 20892, USA; 4 National Institutes of Diabetes & Digestive & Kidney Diseases, National Institutes of Health, Bethesda, MD 20892, USA; 5 Department of Pathology, MedStar Washington Hospital Center, Washington, DC 20010, USA; 6 Section of Endocrinology, MedStar Washington Hospital Center, Washington, DC 20010, USA; 7 Department of Surgery, Georgetown University, Washington, DC 20007, USA; 8 Department of Surgery, MedStar Washington Hospital Center, Washington, DC 20010, USA

**Keywords:** biomarkers, metastases, papillary thyroid cancer, prognosis, progression, recurrence, *SYT12*

## Abstract

**Aim:**

To investigate biomarkers for predicting papillary thyroid cancer outcomes.

**Materials & methods:**

The expression of biomarkers (*ITGA2, SYT12* and *CDH3*) was studied in a prospective cohort of patients with papillary thyroid cancer. Three outcomes of initial metastases, baseline status and longitudinal status were analyzed and correlated with the biomarkers.

**Results:**

*SYT12* provided the best prediction of initial metastasis (sensitivity: 72%; specificity: 54%). *SYT12* had the highest accuracy for predicting longitudinal status (sensitivity: 100%; specificity: 47%). The best performance for longitudinal status resulted from combining *SYT12* with American Thyroid Association risk stratification, with sensitivity and specificity of 88 and 73%, respectively.

**Conclusion:**

*SYT12* has some prognostic significance in papillary thyroid cancer. Further validation studies in larger populations are warranted.

## Lay abstract

Survival and disease recurrence after diagnosis of papillary thyroid cancer is imperfectly predicted by commonly used ‘staging systems’ based mostly on the characteristics of the tumor such as size and location of spread. Also, these staging systems are generally designed to predict survival. Other characteristics, such as gene mutations may be helpful in predicting whether thyroid cancer will behave aggressively or not. This study investigates three genes associated with thyroid tumors and shows that expression of one of them, synaptotagmin 12 (*SYT12*), tends to predict greater progression of disease in patients with papillary thyroid cancer.

Differentiated thyroid cancer (DTC) is one of the most rapidly increasing cancers in the USA. For the period 2007–2011, the largest annual increases in cancer incidence were for cancers of the thyroid (5.3 and 4.5% in men and women, respectively) [1]. Death rates increased by 1.6% in men and 0.8% in women, at a time when the death rates from some other malignancies were decreasing. Papillary thyroid cancer (PTC) is the most prevalent histological thyroid cancer subtype, accounting for at least 80% of all cases [2–4]. DTC prognosis can be estimated based on traditional staging systems that estimate risk of death from thyroid malignancy. According to the seventh edition of the American Joint Cancer Committee/Union Internationale Contre le Cancer staging system (tumor, nodes, metastases [TNM]), patients can be classified as stage I, II, III or IV, with risk of death successively increasing with advancing stage [5,6]. Another staging system with excellent performance is the National Thyroid Cancer Treatment Cooperative Study Group staging system (NTCTCSG) in which survival rates for the four successive stages are better separated compared with other staging systems [2,4,6,7].

With the death rates from DTC typically being relatively low, it is generally more useful for clinicians to be able to estimate recurrence risks, rather than risk of death. The American Thyroid Association (ATA) risk stratification system was developed to better define risk of recurrence as low, intermediate or high [5]. Other systems incorporate algorithms to restratify patients once their initial response to their surgery and radioactive iodine (RAI) therapy has been assessed [8]. Despite use of these clinicopathologic staging systems, DTC can often have an unpredictably either less or more aggressive course than had been anticipated.

These challenges to accurate prognostication and confident prediction of aggressive presentation and recurrence in DTC occur because a cascade of genetic, molecular and cellular mechanisms are involved in the transition from differentiated to undifferentiated thyroid tumor [9]. The wide spectrum of DTC behavior has led to a search for molecular markers that may refine risk stratification. Examples of useful markers for PTC are the presence of *BRAF* V600E mutations, TERT C228T mutations and coexistent *BRAF–TERT* mutations [10]. MicroRNAs (miRNAs) have also been used as prognostic indicators in DTC [11]. Three miRNAs (miR-146b, miR-221 and miR-222) have consistently been found to be overexpressed in PTC tissue, compared with normal thyroid tissue, and also appear to confer high-risk features.

The staging systems that are discussed above may not, alone, be enough to accurately predict the prognostic outcome of DTC. In this prospective pilot study, we sought to identify molecular targets that would aid in predicting prognosis of PTC, along with the ATA risk stratification system. A set of molecular markers was initially selected based on their differential expression in a training set of PTC tissue samples and finally a set of three gene markers (*ITGA2, SYT12* and *CDH3*) was selected and tested for prognostic significance in an independent PTC cohort. To our knowledge, *ITGA2, SYT12* and *CDH3* have not been investigated prospectively as biomarkers for predicting disease progression in patients with PTC.

## Materials & methods

### Discovery component

#### Initial selection of 15 biomarkers

We used gene expression profiling generated by high-throughput platform Gene Expression Omnibus (www.ncbi.nlm.nih.gov/geo) to screen for molecular markers for PTC. Initially, screening of differentially expressed genes in thyroid carcinoma tissues was carried out by manually querying publicly available data at the Gene Expression Omnibus database under the dataset series GSE3678 (www.ncbi.nlm.nih.gov/geo/query/acc.cgi?acc=GSE3678), (samples: GSM85215 thyroid_normal_1, GSM85216 thyroid_normal_2, GSM85217 thyroid_normal_3, GSM85218 thyroid_normal_4, GSM85219 thyroid_normal_5, GSM85220 thyroid_normal_6, GSM85221 thyroid_normal_7, GSM85222 thyroid_tumor_1, GSM85223 thyroid_tumor_2, GSM85224 thyroid_tumor_3, GSM85225 thyroid_tumor_4, GSM85226 thyroid_tumor_5, GSM85227 thyroid_tumor_6 and GSM85228 thyroid_tumor_7). A total of 15 genes were selected (see below). These included genes from this data series whose selection were based on differential expressions between normal and tumor tissue. Other genes of interest based on available laboratory data and primer availability were also chosen.

#### Use of markers in exploratory or training set of 12 tissue samples

**Figure 1.**
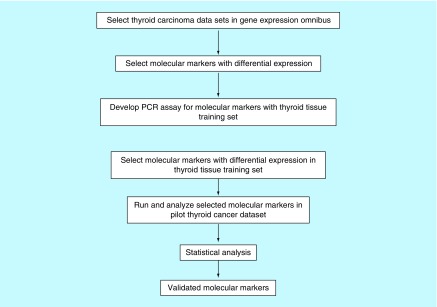
**Flowchart of the method of selecting molecular markers for testing in the training and validation studies.**

These 15 genes (*IPCEF1, FN1, ITGA2, TPO, SYT12, GPM6A, DIO1, CRABP1, STRA6, TFF3, TM7SF4, OTOS, CDH3, DTX4* and *TACSTD2*) were analyzed for expression by qPCR in our exploratory set of 12 normal and tumor thyroid samples obtained from the Georgetown University Tumor Bank. The details of primers used in the mRNA expression studies and the size of the fragment obtained are shown in Table 1. The annealing temperature was 60°C. Patient outcomes, including documentation of recurrence and death, were available for a subset of these tumors. Genes that showed greater than twofold differential expression in the thyroid tumors compared with the normal thyroid tissue, or that showed greater than twofold differential expression in tumors from patients who were known to have poor outcomes such as recurrence or death were selected for further analysis in our independent prospective study. A set of three gene markers (*ITGA2, SYT12* and *CDH3*) were selected on the basis of their differential expression and assayed for prognostic significance in the independent cohort. A brief flowchart of the method used in this study for selecting molecular markers for outcomes in PTC is presented in Figure 1.

**Table 1.** **Details of mRNA expression studies.**

**Primer name**	**Sequence**	**Size of fragment (bases)**
IPCEF1Fwd	aggggtcgtcactgtactgg	184

IPCEF1Rev	cacacgttcatttcctgcac	184

FN1Fwd	accaacctacggatgactcg	230

FN1Rev	gctcatcatctggccatttt	230

ITGA2Fwd	gggcattgaaaacactcgat	183

ITGA2Rev	tcggatcccaagattttctg	183

TPOFwd	tggagaagcactccctgtct	209

TPORev	gtgcacaaagtccccattct	209

SYT12Fwd	agagcctgcggttttctgta	248

SYT12Rev	gtcgttggtccagatgaggt	248

GPM6AFwd	tgagatggcaagaactgctg	238

GPM6ARev	Ccaggccaacatgaaaagat	238

DIO1Fwd	agccacgacaactggatacc	160

DIO1Rev	actcccaaatgttgcacctc	160

CRABP1Fwd	caggacggggatcagttcta	238

CRABP1Rev	cgccaaacgtcaggataagt	238

STRA6Fwd	actggtgacacacaggacca	188

STRA6Rev	tggttcccaggaagaagatg	188

TFF3Fwd	ctccagctctgctgaggagt	164

TFF3Rev	gcttgaaacaccaaggcact	164

TM7SF4Fwd	cacttgaaactgcacggaga	175

TM7SF4Rev	aggacaacagtcccagcatc	175

OTOSFwd	ctgtgcaggaggaaggagac	197

OTOSRev	ctcctgatagggaacgtgga	197

CDH3Fwd	aacctccacagccaccatag	181

CDH3Rev	gtctctcaggatgcggtagc	181

DTX4Fwd	ggagactgcaggacaggaag	233

DTX4Rev	tacccagcaccaaaaactcc	233

TACSTD2Fwd	gatttcggtatcgtcccaga	201

TACSTD2Rev	ggaccgaaaggggatacatt	201

### Validation or prospective component

#### Design of independent study

In this independent study, we examined biomarkers that would aid in prognosis of PTC, along with the ATA risk stratification system. Patients who had been diagnosed with PTC on the basis of a fine needle aspiration biopsy consistent with PTC (Bethesda category VI), or on the basis of a biopsy that was suspicious for malignancy (Bethesda category V) and felt by the cytopathologist to most likely represent PTC and who were scheduled for surgery at MedStar Washington Hospital Center were recruited for this prospective study. Total thyroidectomy was routinely performed during this time period; central and lateral lymph node dissection was performed at the discretion of the surgeon. Patients were simply recruited sequentially and no patient with PTC was excluded. The study was approved by the Joint Oncology Institutional Review Board at Georgetown University and MedStar Washington Hospital Center. Patients were given information about the study by their surgeon. Interested patients signed a written consent form to allow removal of a small portion of both their tumor and normal thyroid tissue, and also permit review of their medical records using MedStar's Electronic Medical Record System. At the time of their thyroidectomy, approximately 1-gm pieces of tissue were removed from the patient's tumor and from uninvolved thyroid tissue by the study pathologist and rapidly frozen. Frozen tissue was immediately transported to the NIH for RNA extraction. The participant's records were reviewed after their initial treatment with surgery and RAI was complete.

The records were also reviewed on an annual basis for at least 4 years, and in a minority of cases for up to 5 years, to determine whether any additional treatment had been administered and what the patient's disease status was. Initial disease status was based on a combination of surgical pathology, results of postoperative RAI scanning and postoperative serum thyroglobulin. Disease status at follow-up was classified as no evidence of disease, biochemical evidence of disease only, regression of disease, stable disease or progression of disease. This status was assigned by one physician based on the results of serum thyroglobulin measurements, thyroglobulin antibody measurements, cervical ultrasonography, 3D imaging (CT scanning, MRI scanning), nuclear medicine studies (RAI whole body scanning and PET scanning) and results of biopsies or additional surgery. A thyroglobulin level of <0.1–0.5 ng/ml was considered undetectable, depending on the assay used. A rising thyroglobulin, or a rising thyroglobulin antibody level if the patient had thyroglobulin antibodies present, was defined as ‘biochemical disease only’ if no structural disease was identified. The physician was blinded to the results of *ITGA2, SYT12* and *CDH3* mRNA analysis until these results were finalized. The surgical, nuclear medicine and endocrinology care received by patients enrolled in the study was that recommended by their healthcare team at MedStar Washington Hospital Center and was unaffected by study participation.

#### Characteristics of participants in independent study

A total of 40 patients were recruited for the independent study during the period 2010–2012. Two patients were ineligible because of a finding of benign thyroid disease and metastatic lung cancer to the thyroid, respectively. Both of these patients had initial biopsies showing suspicious for malignancy rather than PTC. Thus 38 patients were confirmed to have PTC and these patients were monitored for 4–5 years. The initial demographic, PTC and treatment characteristics of the 38 patients are shown in Table 2. Other tumor characteristics included 66% with disease-affecting cervical lymph nodes, 39% with extra-thyroidal extension of their PTC, 63% with multifocal PTC and 11% with distant metastases. None of the histologic variants of PTC were minimally invasive follicular variants. All patients received a total thyroidectomy and 92% received radioiodine (RAI), as was typical for managing DTC at Washington Hospital Center during this time period. The average RAI treatment activity administered was 133 mCi (range: 30–394 mCi). Using the TNM staging system, 61% of patients had stage I disease, 13% had stage II disease, 15% had stage III disease and 11% had stage IV disease. Using the ATA risk stratification system, 47% had low-risk disease, 42% had intermediate-risk disease and 10% had high-risk disease. Five patients (13%) within the cohort had positive thyroglobulin antibodies.

**Table 2.** **Initial characteristics of patients enrolled in prospective study.**

**Patient and tumor characteristics (n = 38)**	**Continuous variables**	

	***Mean***	***SD***
Age (years)	45.5	13.8

Tumor size (largest focus) (cm)	1.9	0.7

Multifocality: number of foci	3	2.9

Number of cervical lymph nodes	7	15

Treatment activity of radioiodine (mCi)	133	79

	**Categorical variables**	
	***Number***	***Percentage***
***Sex***		
– Female	34	89
– Male	4	11
***Total thyroidectomy***		
– Yes	38	100
***DTC histology***		
– Papillary	27	71
– Variants, papillary	11	29
***Cervical node involvement***		
– Yes	25	66
– No	13	34
***Extrathyroidal extension***		
– Yes	15	39
– No	23	61
***Multifocality***		
– Yes	24	63
– No	14	37
***Distant metastases***		
– Yes	4	11
– No	34	89
***Radioiodine therapy***		
– Yes	35	92
– No	3	8
***TNM stage***		
– I	23	61
– II	5	13
– III	6	15
– IV	4	11
***NTCTCSG stage***		
– I	22	58
– II	5	13
– III	9	24
– IV	2	5

DTC: Differentiated thyroid cancer; NTCTCSG: National Thyroid Cancer Treatment Cooperative Study Group; TNM: Tumor, nodes, metastases.

#### mRNA expression studies in 38 resected thyroid tumors from independent cohort

*ITGA2, SYT12* and *CDH3* mRNAs were determined in resected thyroid tumors as follows.

Normal and tumor tissue samples were homogenized using a polypropylene disposable mortar and pestle (Thermo Fisher Scientific, MA, USA) in TRIzol reagent (Invitrogen, CA, USA) and total RNA was extracted. A total of 200 ng of total RNA was reverse-transcribed into complementary DNA using a first strand synthesis kit (Roche, CA, USA) with random hexamers. Complementary DNA generated from the RNA was subjected to real-time quantitative PCR (qPCR) for *ITGA2, SYT12* and *CDH3* expression using a Fast SYBRxGreen Master Mix PCR kit (Applied Biosystems, CA, USA) under the following cycling conditions: initial denaturation for 5 min at 95°C, followed by 40 cycles of 15 s at 95°C and 60 s at 62°C. The PCR reaction was followed by a melting curve program (60–95°C) with a heating rate of 0.1°C per second and a continuous fluorescence measurement and a cooling program at 40°C. Reactions were performed using an ABI PRISM 7500 Sequence Detector (Applied Biosystems). Fluorescence signals were analyzed using SDS 1.9.1 software (Applied Biosystems). The primers used are described earlier.

The relative amounts of biomarker mRNAs (*ITGA2, SYT12* and *CDH3*) were normalized to the housekeeping gene 18S. Fold changes were calculated using the equation 2 − ΔΔC_T_, ΔΔC_T_ = [C_T_ (biomarker gene) − C_T_ (18S)] test − [C_T_ (biomarker) − C_T_ (18S)] calibrator. The relative expression level of the biomarkers in each sample was evaluated as the relative fold change in log 2 scale. The T/N ratio was derived from the expression level of the biomarkers in the tumor tissue (T) compared with that of the normal thyroid tissue (N). All the samples were analyzed in triplicate.

#### Risk classification & outcome variables used for participants in independent study

Patients were classified as to whether they were at low, intermediate or high risk for recurrence based on the 2016 ATA risk stratification system [5].

**Figure 2.**
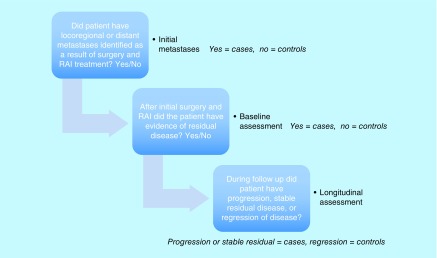
**Definition of the dichotomized outcomes of initial metastases, baseline status and longitudinal status used in the prospective cohort.**

Because of the small sample size, disease status was also consolidated into three binary outcome variables (initial metastasis, baseline status and longitudinal status) (Figure 2) that were examined in relation to the *ITGA2, SYT12* and *CDH3* mRNA biomarkers.

##### Initial metastasis

The extent of metastases at initial presentation was assessed using information from the surgical pathology and results of initial therapy with RAI. This variable was dichotomized as no locoregional or distant metastasis (controls) versus either locoregional or distance metastasis (cases).

##### Baseline status

Patients were assessed at approximately 2 months after their initial treatment with surgery and radioactive iodine therapy for evidence of disease. Patients who showed no evidence of disease after initial treatment were the controls, and the cases included patients with remaining biochemical disease, nodal disease or other metastases (e.g., pulmonary or skeletal) after initial treatment.

##### Longitudinal status

This variable, indicating whether a patient had progression or regression of disease, was generated from the annual evaluations of disease status over the 4- to 5-year follow-up. The regressors included patients with no evidence of disease at the baseline assessment who remained nondiseased over time; and patients with evidence of disease at the baseline who regressed to nondiseased. The progressors included patients with no evidence of disease at the baseline who progressed to diseased; and patients with evidence of disease at the baseline whose disease further progressed or remained stable.

#### Statistical analysis for independent study

Summary statistics were tabulated for the binary disease outcomes and biomarkers. A scatterplot matrix visualized the correlation structure of the three biomarkers. Using a continuous scale for the biomarkers, a receiver operating characteristic (ROC) curve was plotted for each of the biomarkers and outcomes. As a measure of the diagnostic/prognostic accuracy, the area under ROC curve (AUC) was calculated, and tested to determine if the AUC was greater than 0.5 using the Wilcoxon rank sum test. The significance level was 0.05.

To study the sensitivity (Se) and specificity (Sp) of the biomarkers, we dichotomized them using prespecified cut-off points: *ITGA2, SYT12* and *CDH3* mRNA were positive if their fold expression was greater than or equal to 2, 4 and 10, respectively. Se and Sp were calculated, as well as their exact 95% CI. As Se or Sp cannot be examined alone, but are better viewed together as a tradeoff between false-positive and false-negative errors, we also calculated Youden's index [12]. This is a summary measure of Se and Sp, defined as Se + Sp -1. A larger Youden's index represents better accuracy of a binary test.

A combination of the several biomarkers can often improve diagnosis with, for example, multiple positive biomarkers being a strong indication of disease. Therefore, we counted the number of positive biomarkers (ranging from 0 to 3) for each patient, and then defined a ‘positive result’ of the combined markers in two ways: two or more markers positive and all three markers positive. The Se, Sp and Youden's index of the combinations were evaluated.

Since ATA risk categories are a commonly used clinical assessment of the possibility of recurrence, we also estimated the accuracy of ATA risk stratification in predicting the longitudinal outcome. After dichotomizing ATA risk as low versus intermediate/high, we calculated its Se, Sp and Youden's index for predicting the longitudinal disease outcome, and qualitatively compared them with the accuracy measures of the three biomarkers.

All the statistical analyses were conducted in R version 3.1.3.

## Results

### Characteristics & outcomes of the PTC patient cohort

The characteristics of the cohort are described in section 2b of the ‘Materials & methods’. Our study cohort received care at MedStar Washington Hospital Center from either their endocrinologist alone, or from their endocrinologist and other specialists. A total of 76% of patients had  four full years of follow-up; the remaining 24% of patients had 5 years of follow-up (Table 3 and Table 4). A total of 66% of patients had locoregional or distant metastases at presentation, 34% still had evidence of disease after their initial treatment (Table 3). At the end of 4 years of follow-up, 30/38 (79%) of patients had no evidence of PTC, whereas 8/38 (21%) had residual disease that was stable in five patients and progressive in three patients. The eight patients that had residual disease had disease present in cervical lymph nodes, the lungs, the skeleton and the liver (Table 4). No patient died during the 4–5 years of follow-up. The three outcome variables of initial metastases, baseline status and longitudinal status and ATA risk category are summarized as percentages in Table 5 and Table 6, respectively.

**Table 3.** **Longitudinal follow-up of the patients in the prospective study: DTC status.**

**Category**	**Number of patients in each category**					

**DTC status^†^**	**No evidence of disease**	**Biochemical disease only**	**Residual structural disease**	**Regression of disease**	**Stable disease**	**Progression of disease**
Baseline status^‡^	25	4	9	N/A	N/A	N/A

1-year follow-up	25	2	10	2	9	2

2-year follow-up	27	1	8	3	6	1

3-year follow-up	29	0	7	2	6	1

4-year follow-up	30	0	8	0	5	3

5-year follow-up	6	0	3	0	2	1

^†^n = 38 patients for initial status and years 1–4, n = 9 for fifth year of follow-up, may be more than one disease status per patient.

^‡^Baseline disease status after initial treatment was assessed based on a combination of surgical pathology, results of postoperative radioiodine scanning and postoperative thyroglobulin measurement at approximately 2 months after surgery.

DTC: Differentiated thyroid cancer.

**Table 4.** **Longitudinal follow-up of the patients in the prospective study: disease location.**

**Category**	**Number of patients in each category**				

**Disease location^†^**	**None known**	**Cervical**	**Pulmonary**	**Skeletal**	**Hepatic**
Baseline status^‡^	29	5	4	0	0

1-year follow-up	28	5	5	0	0

2-year follow-up	28	4	4	0	0

3-year follow-up	29	3	4	0	1

4-year follow-up	30	5	4	1	1

5-year follow-up	6	2	1	0	1

^†^n = 38 patients for initial status and years 1–4, n = 9 for fifth year of follow-up, may be more than one disease location per patient.

^‡^Baseline disease status after initial treatment was assessed based on a combination of surgical pathology, results of postoperative radioiodine scanning and postoperative thyroglobulin measurement at approximately 2 months after surgery.

**Table 5.** **Thyroid cancer status for patients in the prospective study.**

**Disease status**	**Summary statistics using count (percentage)**
***Initial metastasis^†^***	

No	13 (34.2%)

Yes	25 (65.8%)

***Baseline status^‡^***	

No evidence of disease	25 (65.8%)

Evidence of disease	13 (34.2%)

***Longitudinal status (Fourth year)^§^***	

Regression	30 (78.9%)

Progression	8 (21.1%)

^†^Initial metastasis = 0 if none; 1 if metastasis is documented.

^‡^Baseline status = 0 if no disease, 1 if any disease still present.

^§^Longitudinal status = 0 if initial status = 1 and regressed to NED, or initial status = 0 and stayed at NED (those who recovered); = 1 if initial status = 1 and remained stable/progressed, or initial status = 0 and progressed to diseased (those who did not recover).

NED: No evidence of disease.

**Table 6.** **ATA risk category for patients in the prospective study.**

**ATA risk category**	**Summary statistics using count (percentage)**
Low	18 (47.4%)

Intermediate	16 (42.1%)

High	4 (10.5%)

ATA: American Thyroid Association.

### mRNA expression studies

#### Total RNA quality

High-quality RNA was prepared from tissue samples of patients, as reported in the ‘Material & methods’. The RINs (RNA integrity) were between 9.2 ± 0.5; the ratio of the absorbance at 260 and 280 nm was 1.98 ± 0.06 for all samples.

#### Expression of ITGA2, SYT12 & CDH3 mRNA in PTC

As previously described, we computationally selected 15 genes (*IPCEF1, FN1, ITGA2, TPO, SYT12, GPM6A, DIO1, CRABP1, STRA6, TFF3, TM7SF4, OTOS, CDH3, DTX4, TACSTD2*) that were differentially regulated in thyroid tumors. After real-time PCR screening analysis of these 15 genes in the exploratory PTC tissue samples (training set), we found three genes (*ITGA2, SYT12* and *CDH3*) that had significant differential expression in PTC tissues compared with normal thyroid tissues and in aggressive tumors compared with more indolent ones. Therefore, we focused on these transcripts that were highly expressed in the PTC training set in order to potentially improve our test accuracy in this independent study. The expression of these three genes was therefore determined in our prospective cohort of 38 patients with PTC. The log 2 transformed fold changes in these genes in the tumor tissue of each patient are shown in Table 7, with the fold changes in *TPO* and *CRABP1* shown for comparison. The median change in expression was 5.2-fold (range: 0.19- to 71-fold; p < 0.001), eightfold (range: 0.1- to 507-fold; p < 0.003) and 15-fold (range: 0.1- to 323-fold; p < 0.001) for *ITGA2, SYT12* and *CDH3*, respectively. Fold change cut-off levels with the optimum diagnostic efficiency derived from the ROC curves were two for *ITGA2*, four for *SYT12* and ten for *CDH3*.

**Table 7.** **Fold change in mRNA expression in tumor tissue compared with matched normal tissue, log 2 transformed and normalized to 18s rRNA for each patient in the prospective cohort.**

**Patient #**	**Genes**				

	***SYT12***	***ITGA2***	***CDH3***	***TPO***	***CRABP1***
1	179.15	3.64	29.45	0.02	0.41

2	19.03	4.74	15.67	0.25	0.26

3	4.58	2.96	14.72	0.08	0.09

4	7.57	2.00	1.66	3.41	1.99

5	4.86	9.51	2.48	0.03	0.00

6	10.52	7.70	1.20	0.56	0.23

7	0.06	0.19	3.11	0.67	0.57

8	48.50	5.66	49.69	0.13	0.08

9	20.32	3.51	18.96	0.07	0.03

10	8.00	13.88	72.00	4.89	1.31

11	374.81	28.94	51.80	0.13	0.27

12	1.66	3.33	1.99	0.53	1.63

13	16.91	54.00	93.70	0.01	0.12

14	47.84	7.24	54.57	0.02	0.01

15	48.17	16.97	92.09	0.01	0.02

16	2.43	2.58	13.69	0.36	0.02

17	2.32	0.69	3.64	0.30	0.46

18	506.70	27.38	100.78	0.13	0.03

19	63.78	19.56	67.88	0.00	0.00

20	137.19	17.94	86.52	1.93	0.62

21	1.02	1.78	3.73	0.55	0.47

22	203.66	36.13	70.77	0.05	0.09

23	1.01	1.50	2.21	1.60	0.92

24	10.23	9.68	14.98	0.03	0.01

25	58.28	42.81	314.08	0.22	0.26

26	81.01	28.84	17.63	0.03	0.01

27	69.31	50.04	116.97	0.68	0.30

28	0.67	0.72	0.52	0.00	0.55

29	1.11	0.99	12.08	0.51	0.24

30	74.80	70.77	160.34	1.45	0.32

31	0.45	1.32	1.52	15.51	1.97

32	0.30	11.55	4.44	3.05	2.32

33	0.35	9.03	1.81	3.04	66.26

34	0.30	3.19	32.56	2.15	0.27

35	28.64	5.22	322.91	0.01	0.01

36	1.45	1.51	0.10	0.00	1.56

37	0.84	2.71	4.17	2.44	2.11

38	2.69	0.36	1.78	1.00	0.92

### Accuracy of the biomarkers

The biomarker data are summarized in Table 8, with percentages reported for categorical variables and mean (SD) for continuous variables. The log 2 transformed fold change in each of the biomarkers, and the percentage of patients who had 0–3 positive biomarkers is shown.

**Table 8.** **Biomarker summary statistics: median (interquartile range) for fold change in biomarkers after log 2 transformation and count (percentage) for number of biomarkers.**

**Biomarkers**	**Summary statistics**
*ITGA2^†^*	6.4 (2.6–19.2)

*SYT12^‡^*	10.4 (1.5–55.8)

*CDH3^§^*	16.7 (3.2–71.7)

***Number of positive markers***	

0	7 (18.4%)

1	6 (15.8%)

2	4 (10.5%)

3	21 (55.3%)

^†^ITGA2 is positive if greater than 2.

^‡^SYT12 is positive if greater than 4.

^§^CDH3 is positive if greater than 10.

**Figure 3.**
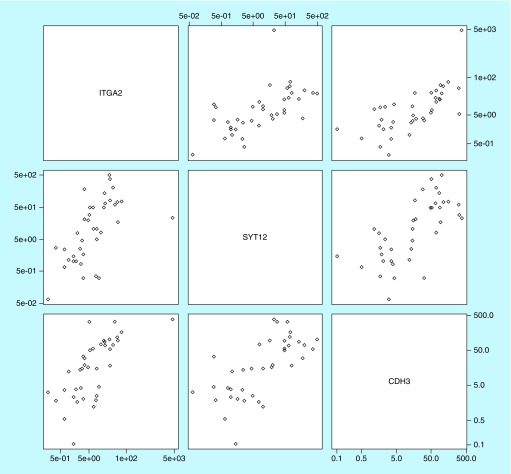
**Scatterplot matrix for *ITGA2, SYT12* and *CDH3* visualizing the correlation structure of the three biomarkers using the relative fold change in log 2 scale in the marker in tumor tissue compared with normal thyroid tissue.** Correlation coefficients are between 0.60 and 0.69.

**Figure 4.**
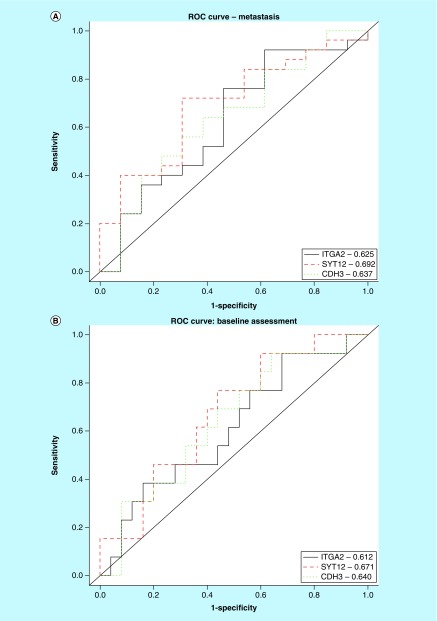

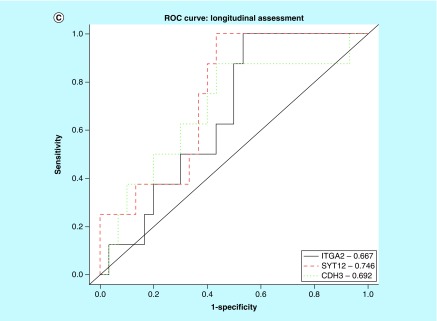
**ROC curves for *ITGA2, SYT12* and *CDH3* as predictors of PTC outcomes.** **(A)** Area under ROC curves for *ITGA2, SYT12* and *CDH3* demonstrating performance in diagnosing PTC metastasis at initial presentation (ROC values indicated within box). **(B)** Area under ROC curves for *ITGA2, SYT12* and *CDH3* demonstrating performance in predicting baseline status of residual disease after initial treatment (ROC values indicated within box). **(C)** Area under ROC curves for *ITGA2, SYT12* and *CDH3* demonstrating performance in predicting longitudinal status of disease progression after initial treatment (ROC values indicated within box). PTC: Papillary thyroid cancer; ROC: Receiver operating characteristic.

The three markers were all positively correlated with each other (Figure 3), with correlation coefficients between 0.60 and 0.69. Figure 4A–C show plotted area under the ROC curves for *ITGA2, SYT12* and *CDH3* mRNA in association with the three outcomes: initial metastasis, baseline status and longitudinal status. Table 9 shows the area under these ROC curves (AUC) with the 95% CI. *SYT12* had the highest diagnostic accuracy for all three outcomes, with AUCs ranging from 0.671 to 0.746 (p-values: 0.017–0.045). The Wilcoxon rank sum test suggested that the AUCs for *SYT12* were significantly larger than 0.5. *CDH3* had borderline significant AUCs between 0.637 and 0.692, and p-values ranging from 0.052 to 0.089. *ITGA2* only had marginal significant for prediction of longitudinal disease progression. The combination of all three biomarkers was not superior to the performance of *SYT12* alone.

**Table 9.** **Area under ROC curves for biomarker prediction.**

**Biomarker**	**Area under ROC curves X 100 (95% confidence interval)**		

	**Initial metastasis^†^**	**Baseline status^‡^**	**Longitudinal status^§^**
*ITGA2*	62.5 (42.4, 82.5)	61.2 (42.0, 80.5)	66.7 (48.4, 84.9)

*SYT12*	**69.2 (51.5, 87.0)**	67.1 (49.3, 84.9)	**74.6 (57.6, 91.6)**

*CDH3*	63.7 (44.2, 83.2)	64.0 (45.3, 82.7)	69.2 (46.6, 91.7)

Bold indicates the best performance.

^†^Initial metastasis = 0 if none; 1 if metastasis is documented.

^‡^Baseline status = 0 if no disease, 1 if any disease is still present.

^§^Longitudinal status = 0 if initial status = 1 and regressed to NED, or initial status = 0 and stayed at NED (those who recovered); = 1 if initial status = 1 and remained stable/progressed, or initial status = 0 and progressed to diseased (those who did not recover).

NED: No evidence of disease; ROC: Receiver operating characteristic.

See Figure 4A–C for actual ROC curves.

Table 10 shows the diagnostic accuracy of the biomarkers in predicting the dichotomized outcomes, and reports their Se and Sp with the exact 95% CI. *SYT12* had the best performance among the three markers for diagnosing initial metastasis and for predicting disease progression over time, with Youden's indices of 0.26 and 0.47, respectively. *ITGA2* had a Youden's index of 0.24 in predicting baseline residual disease. Also shown in Table 10, is the performance of the combined biomarkers based on the number of positive markers. Using a cut-off point of 2 or more positive biomarkers, the diagnostic accuracy was the highest for baseline and longitudinal status, with a Youden's index of 0.29 and 0.43, respectively. Using all three positive biomarkers the accuracy for predicting initial metastasis was similar to that of *SYT12* alone, with a Youden's index of 0.26.

**Table 10.** **Sensitivity and specificity of the biomarkers in diagnosis and prediction of outcomes in the prospective cohort of patients.**

	**Sensitivity (95% CI)**	**Specificity (95% CI)**	**Youden's index**
***Outcome: initial metastasis***			

*ITGA2*^†^	0.84 (0.64, 0.95)	0.38 (0.14, 0.68)	0.22

***SYT12***^‡^	**0.72 (0.51, 0.88)**	**0.54 (0.25, 0.81)**	**0.26**

*CDH3*^§^	0.68 (0.46, 0.85)	0.46 (0.19, 0.75)	0.14

# Positive markers ≥2	0.72 (0.51, 0.88)	0.46 (0.19, 0.75)	0.18

**# Positive markers = 3**	**0.64 (0.43, 0.82)**	**0.62 (0.32, 0.86)**	**0.26**

***Outcome: baseline status***			

***ITGA2***	**0.92 (0.64, 1.00)**	**0.32 (0.15, 0.54)**	**0.24**

*SYT12*	0.77 (0.46, 0.95)	0.44 (0.24, 0.65)	0.21

*CDH3*	0.77 (0.46, 0.95)	0.44 (0.24, 0.65)	0.21

**# Positive markers ≥2**	**0.85 (0.55, 0.98)**	**0.44 (0.24, 0.65)**	**0.29**

# Positive markers = 3	0.69 (0.39, 0.91)	0.52 (0.31, 0.72)	0.21

***Outcome: longitudinal status***			

*ITGA2*	1.00 (0.52, 1.00)	0.30 (0.15, 0.49)	0.30

***SYT12***	**1.00 (0.52, 1.00)**	**0.47 (0.28, 0.66)**	**0.47**

*CDH3*	0.88 (0.47, 1.00)	0.43 (0.25, 0.63)	0.31

# Positive markers ≥2	1.00 (0.52, 1.00)	0.43 (0.25, 0.63)	0.43

# Positive markers = 3	0.88 (0.47, 1.00)	0.53 (0.34, 0.72)	0.41

ATA risk (intermediate/high)	0.88 (0.47, 1.00)	0.57 (0.37, 0.75)	0.44

**ATA risk (intermediate/high) and *SYT12* positive**	**0.88 (0.47, 1.00)**	**0.73 (0.54, 0.88)**	**0.61**

ATA risk (intermediate/high) and # positive markers ≥2	0.88 (0.47, 1.00)	0.70 (0.51, 0.85)	0.57

ATA risk (intermediate/high) and # positive markers = 3	0.75 (0.35, 0.97)	0.77 (0.58, 0.90)	0.52

^†^ *ITGA2* is positive if greater than 2.

^‡^ *SYT12* is positive if greater than 4.

^§^ *CDH3* is positive if greater than 10.

Note: Exact confidence intervals were calculated due to small counts in the 2 by 2 table.

Bold indicates highest Youden's index for each outcome.

ATA: American Thyroid Association.

ATA risk category was also used to predict longitudinal outcomes and this had an Se of 88%, an Sp of 57% and a Youden's index of 0.44. When combined with two or more positive markers, the Se was unchanged, but the Sp improved to 70%, with a Youden's index of 0.57. When combined with all three markers being positive, Se declined to 75%, although Sp increased to 77%, with a Youden's index of 0.52. However, the best performance was obtained using a combination of ATA risk category and *SYT12* with an Se of 88%, an Sp of 73% and a Youden's index of 0.61.

## Discussion

In this research study, we interrogated 15 mRNA that were associated with thyroid tumors and had differential expression in a high-throughput expression dataset. We performed an initial qPCR screening to further determine how many of these 15 genes would be a useful prognostic biomarker for PTC, and found that *ITGA2, SYT12* and *CDH3* had significant differential expression in specimens from a tissue bank.

Biomarkers were then validated in resected thyroid tumors in a prospective study. Our current results indicate that high levels of these three genes tend to be associated with presentation with metastases, presence of residual disease after initial treatment and short-term longitudinal outcomes. Of these, *SYT12* had greatest predictive ability based on the ROC curve. When examining Se and Sp, *SYT12* was also the single-best predictor of initial metastases and longitudinal outcome. However, *ITGA2* or two or more positive markers provided the best Se, Sp or performance for predicting residual disease after initial treatment. However, all of these markers had relatively low predictive value. The finding that *SYT12* is the best of the three biomarkers for PTC appears to be a congruent with the recent finding that *SYT12* is one of the genes in the 71-gene signature that distinguishes *BRAF*-like tumors from NRAS-like tumors [13]. In addition, *CDH3* gene expression has been found to discriminate PTC from normal thyroid tissue [14], and *ITGA2* gene expression has been associated with PTC and aggressiveness of PTC [15–17].

The *SYT12* gene or synaptotagmin 12 (also known as synaptotagmin-related gene 1 or *SRG1*) encodes a family of proteins involved in the regulation of transmitter release in the nervous system. *SYT12* is rapidly inducible by thyroid hormone and the timing of its expression is consistent with regulation by thyroid hormone during development [18]. *SYT12* is likely a part of a cascade of gene activation induced by thyroid hormone that is critical for CNS organization and development [19]. Regulation by thyroid hormone is not a feature common to all members of the synaptotagmin family, suggesting that *SYT12* may have unique functions among the synaptotagmins. As mentioned above, the *SYT12* gene is also associated with *BRAF*-positive tumors [13].

The *ITGA2* gene encodes alpha-2 integrin, a membrane glycoprotein known as GP Ia, which is expressed in a variety of cell types. Integrins are transmembrane adhesion molecules that mediate cell–cell and cell–extracellular matrix attachment. As a consequence, integrins regulate cell growth, proliferation, migration and apoptosis and, thus, have a potential role in tumor progression and metastasis. *ITGA2* has been shown to be differentially overexpressed in PTC compared with benign thyroid tissue [15,17] and in aggressive compared with nonaggressive PTC [16]. The effect of *ITGA2* seems to be mediated via abherent expression of miRNAs such as miR-107, miR-103 and miR-195. Cadherins, such as *CDH3*, are integral membrane glycoproteins responsible for calcium-dependent cell–cell adhesion. *CDH3* was found to be helpful in discriminating PTC from normal tissue when included in a panel of 19 genes [14]. However, many other molecular profiling studies have failed to implicate the *ITGA2, SYT12* and *CDH3* genes in thyroid tumorigenesis [20–23].

Interestingly, the gene that we found to be the strongest prognosticator, *SYT12*, is one of the genes that is associated with tumors that carry a *BRAF V600E* mutation [13]. *SYT12* is important in brain development and reduction in thyroid hormone levels leads to reduced *SYT12* expression in the fetal brain. Given that low TSH concentrations (presumably accompanied by high thyroid hormone levels) are beneficial for thyroid cancer outcomes [2,3], it could be speculated that the negative impact of *SYT12* is not mediated through high thyroid hormone levels. The worse longitudinal outcomes associated with *SYT12* could be associated with the *BRAF* mutation, or could be speculated to be associated with refractoriness to RAI therapy.

There are several limitations of our study. These include a small sample size in the independent cohort, a short duration of follow-up, conduct of the study prior to the publication of informative data regarding other gene sets and lack of *BRAF* status for these tumors. The small sample size and resultant small number of outcomes necessitated dichotomized outcomes for the purposes of analysis. Ideally, longer than 4 years would be needed to assess disease progression in PTC. This study was approved in 2010 and conducted prior to the publication of databases such as the Cancer Genome Atlas, such that the comparison of these genes sets was not included in the study protocol. Furthermore, *BRAF* status is not available for these tumors. In addition, Washington Hospital Center is a referral center for DTC and the distribution of disease, such as the high percentage of patients with extra-thyroidal spread of their tumor and distant metastases, may not be representative of the general population of patients with PTC. These limitations lead to data that are suggestive, but require validation in a larger dataset or cohort.

In summary, the biomarkers examined in this study may improve PTC risk stratification in several ways. They appear to have better performance in predicting longitudinal outcomes than features of initial presentation. *SYT12* has the best accuracy as a single marker, and may be one of the genes conferring worse outcomes in *BRAF*-positive tumors. It has better Se and lower Sp than ATA risk stratification for longitudinal outcomes. However, the best performance was obtained by combining *SYT12* with ATA risk stratification. *SYT12* and ATA risk stratification did not have perfect concordance, again with *SYT12* having better Se and ATA risk stratification having better Sp. This suggests that these markers may be reflecting different properties of the disease.

## Conclusion

To our knowledge, *ITGA2, SYT12* and *CDH3* have not been prospectively investigated as biomarkers for predicting disease outcomes in patients with PTC. In this study, we recruited a cohort of patients with PTC and determined the levels of *ITGA2, SYT12* and *CDH3* mRNA in resected thyroid tumors. We examined the accuracy of these markers in predicting baseline and prospective disease status. The three computationally selected and experimentally validated biomarkers show some promising results that warrant further investigation in larger studies.

The demonstration that increased expression of these genes has a negative impact on PTC outcomes, which may lead to the future development of additional therapies for PTC. Furthermore, the methodology used in this study can be applied to other carcinomas.

## Future perspective

Prognostication and selection of appropriate treatment are critical aspects of caring for patients with thyroid cancer. Tools to predict PTC outcomes are relatively imperfect and generally survival is more accurately predicted than recurrence. Treatment is often not selectively applied as the factors governing response to treatment are not fully understood. Use of genetic biomarkers, and perhaps among them *SYT12* expression, may allow better prediction of outcomes and more targeted selection of treatment. As more genes involved in thyroid cancer are identified, the challenge will be to determine their role in cancer development and their interaction with each other. *SYT12* appears to be one of the genes associated with the action of *BRAF*.

**Summary points**

- *ITGA2, SYT12* and *CDH3* had differential expression in papillary thyroid cancer (PTC) tumors compared with normal thyroid tissues.
- *ITGA2, SYT12* and *CDH3* generally had differential expression in PTC tumors that were more aggressive versus less aggressive in their presentation and progression.
- *SYT12* predicted initial metastases (the extent of metastases at initial presentation) in a prospective PTC cohort (sensitivity [Se]: 72%; specificity [Sp]: 54%).
- Two or more positive biomarkers predicted baseline status (the presence or absence of disease after initial treatment) in a prospective PTC cohort (Se: 85%; Sp: 44%).
- *SYT12* predicted longitudinal status (the presence or absence of disease during follow-up) in a prospective PTC cohort (Se: 100%; Sp: 47%).
- American Thyroid Association risk category had an Se of 88% and Sp of 57% for predicting longitudinal status in a prospective PTC cohort.
- *SYT12* combined with ATA risk stratification best predicted longitudinal status in a prospective PTC cohort (Se: 88%; Sp: 73%).
